# Neuropeptide GPCRs in neuroendocrinology: the case of activity-dependent neuroprotective protein (ADNP)

**DOI:** 10.3389/fendo.2012.00134

**Published:** 2012-11-16

**Authors:** Illana Gozes

**Affiliations:** The Lily and Avraham Gildor Chair for the Investigation of Growth Factors, Department of Human Molecular Genetics and Biochemistry, Sagol School of Neuroscience, Adams Super Center for Brain Studies, Sackler Faculty of Medicine, Tel Aviv UniversityTel Aviv, Israel

## Vasoactive intestinal peptide (VIP): neuroprotection and synapse formation

This review is a selected overview of the chosen subject. As, I have recently summarized my work on VIP (Gozes, 2008), I will describe here only selected topics focusing on the GPCR connection of the VIP-regulated protein, activity-dependent neuroprotective protein (ADNP) (Gozes, 2007).

In the mid 1980s we were the first to clone the gene encoding the 28 amino acid neuropeptide, vasoactive intestinal peptide (VIP) (Bodner et al., 1985). This allowed us to follow up VIP gene expression in the brain showing developmental increases at the time of postnatal synapse formation and glial expansion (Gozes et al., 1987). Given the increased expression of VIP at the time of synapse formation and glial expansion, we hypothesized a developmental role for the peptide—associated with synaptogenesis and neuroprotection. Follow-up studies by the laboratory of Douglas Brenneman indeed showed that VIP provided neuroprotection through glial cells (Brenneman et al., 1987). We teamed up to show together with the group of Ronald McKay that VIP enhances synapse formation through glial cell activation (Blondel et al., 2000).

Together with the late Frank Baldino, we showed extensive VIP mRNA expression in the brain, for example, in the suprachiasmatic nucleus (SCN) (Card et al., 1988), an area regulating diurnal rhythms. Our further studies utilizing our VIP hybrid antagonist showed that VIP function during development was required for maintenance of diurnal rhythmicity (Gozes et al., 1995). These findings were later confirmed by knocking out either VIP (Loh et al., 2011), or the VIP receptor VPAC2 (Harmar et al., 2002), which brings us to part of the subject of this review, GPCRs.

## VIP receptors

We have recently reviewed the current status of research on VIP receptors (Harmar et al., 2012). In short, VIP and pituitary adenylate cyclase-activating polypeptide (PACAP) are members of a superfamily of structurally related peptide hormones that includes glucagon, glucagon-like peptides, secretin, gastric inhibitory peptide (GIP) and growth hormone-releasing hormone (GHRH). VIP and PACAP exert their actions through three GPCRs—PAC1, VPAC1 and VPAC2—belonging to class B (also referred to as class II, or secretin receptor-like GPCRs). PAC1 receptors are selective for PACAP, whereas VPAC1 and VPAC2 respond to both VIP and PACAP with high affinity (Harmar et al., 2012). As indicated above, VPAC2 was associated with diurnal rhythmicity (Harmar et al., 2002) as well as astrogenesis (Zupan et al., 1998). Other functions, including association with cancer propagation and immunomodulation have been extensively reviewed (as cited above).

## VIP receptors and neuroprotection, our point of view

Prior to the molecular cloning of VIP and PACAP receptors, binding and displacement assays coupled to functional assays suggested high affinity binding for VIP on glial cells (astrocytes)—associated with the release of neuroprotective proteins and low affinity binding to astrocytes, associated with cAMP formation (Gozes et al., 1991). With the available VPAC1, VPAC2, and PAC1 clones (including the alternatively spliced PAC1 receptors), we have used antisense oligodeoxynucleotides specific for each of the then known receptor subtypes to identify receptors involved in neuroprotection. Our data showed that one of the PAC1 splice variants, the hop2-like receptor is a receptor that mediates neuroprotection (Ashur-Fabian et al., 1997). The PAC1 cDNA was cloned from rat cerebral astrocytes. Using genetic manipulation we obtained the hop2 splice variant and expressed it in COS-7 cells. Results showed that VIP bound the cloned hop2 splice variant (Pilzer and Gozes, 2006). Stearyl-neurotensin (6–11) VIP (7–28) (SNH), an antagonist for VIP, was also found to bind hop2. Other studies have shown that this particular antagonist that we have developed (Gozes et al., 1995) binds to all know VIP receptors (Moody et al., 2002). In addition, VIP protected COS-7 cells expressing hop2 from oxidative stress. Parallel assays demonstrated that VIP increased cAMP accumulation in COS-7 cells expressing hop2. These results support the hypothesis that hop2 mediates some of the cytoprotective effects attributed to VIP (Pilzer and Gozes, 2006).

However, the study cited above did not address the requirement for astrocytes for VIP neuroprotection, suggesting additional players.

## VIP stimulate astrocyte secretion of growth factors

We teamed up together with Douglas Brenneman to isolate novel growth factors from VIP-stimulated astrocyte conditioned media. Our first attempt included sequential chromatography coupled to functional assays of neuroprotection, yielding activity-dependent neurotrophic factor (ADNF) (Brenneman and Gozes, 1996). This study took us to three directions: (1) identification of an active peptide site within ADNF—potential future therapeutic; (2) production of antibodies against ADNF as tools for functional studies and (3) use of the antibodies for expression cloning toward the identification of novel ADNF-like molecules, thus cloning ADNP (Bassan et al., 1999). We have further identified ADNP immunoreactivity in astrocyte conditioned medium, which was increased upon exposure to VIP (Furman et al., 2004).

## GPCR involvement in ADNP expression

Using VIP analogues specific for the VPAC1 and the VPAC2 receptors, we discovered that VIP-induced changes in ADNP expression in astrocytes via the VPAC2 receptor. The constitutive synthesis of ADNP and VPAC2 was shown to be age-dependent and increased as the astrocyte culture developed. The VIP-related peptide, PACAP also induced changes in ADNP expression. The apparent change-induced by VIP and PACAP on ADNP expression were developmentally dependent, and while stimulating expression in young astrocytes (Bassan et al., 1999), an inhibition was demonstrated in older cultures suggesting that VIP, PACAP, and the VPAC2 receptor may all contribute to the regulation of ADNP gene expression in the developing astrocyte (Zusev and Gozes, 2004).

Parallel studies by the discoverer of PACAP, the late Akira Arimura, showed that when PACAP38 was added to mouse neuroglial cultures, it induced ADNP mRNA expression in a bimodal fashion at subpico- and nanomolar concentrations with greater response at subpicomolar level. The response was attenuated by a PAC1 receptor antagonist at both concentrations and by a VPAC1 receptor antagonist at nanomolar concentration only. An IP3/PLC inhibitor attenuated the response at both concentrations of PACAP38, but a MAPK inhibitor had no effect. A PKA inhibitor suppressed the response at nanomolar concentration only. The findings suggest that ADNP expression is mediated through multiple receptors and signaling pathways that are regulated by different concentrations of PACAP (Nakamachi et al., 2006) as well as VIP. A further study showed that ADNP-immunoreactive cells in the cerebral cortex were multi-polar-shaped and co-immunostained with the astrocyte marker, glial fibrillary acidic protein (GFAP). ADNP-immunoreactive cells in the cerebellum were found to surround Purkinje cells and showed GFAP immunoreactivity. In contrast, ADNP-immunoreactive cells in the hippocampus and septum were round in shape and co-immunostained with neuron-specific enolase (NSE). Importantly, all of the ADNP-immunopositive cells co-localized with PAC1 immunoreactivity. The observations suggest that ADNP is expressed in both astrocytes and neurons, and that ADNP expression may be regulated in part by PACAP (Nakamachi et al., 2008).

Using bioinformatics analysis, we have identified an ADNP family member which we have named, ADNP2 (Zamostiano et al., 2001; Kushnir et al., 2008).

To evaluate the impact of VIP expression *in vivo* on ADNP and the related protein ADNP2, we examined gene expression in adult wild-type (VIP+/+) and VIP null (VIP−/−) offspring of VIP deficient mothers (VIP+/−) comparing them to wild-type offspring of wild-type mothers. Quantitative real time polymerase chain reaction (PCR) revealed regionally specific reductions of ADNP mRNA in the brains of VIP−/− mice compared with the brains of wild-type offspring of a wild-type mother. ADNP was significantly reduced in the cortex and hypothalamus of VIP−/− mice, but not in the hippocampus or thalamus. ADNP2 exhibited a similar pattern but reached a statistically significant reduction only in the hypothalamus. The RNA transcripts for ADNP and ADNP2 also tended to be reduced in the cortex and hippocampus of the wild-type littermates of the VIP−/− mice, indicating that the VIP genotype of the mother may have had an impact on the ADNP expression of her offspring, regardless of their own VIP genotype. Thus, VIP regulates brain ADNP expression in a regionally specific manner and both maternal and offspring VIP genotype may influence ADNP expression in the brain (Giladi et al., 2007).

## Clinical implications

Recent genetic studies titled: duplications of the neuropeptide receptor gene VPAC2 confer significant risk for schizophrenia implicated the VPAC2 receptor in susceptibility to schizophrenia. Further studies implicated the PAC1 receptor and PACAP in post-traumatic stress disorder as recently reviewed (Harmar et al., 2012).

In this respect, we found deregulation of ADNP/ADNP2 in the postmortem hippocampus of schizophrenia patients (Dresner et al., 2011) and correlated with disease duration.

Other findings associated reduced ADNP expression to the progression of multiple sclerosis and increased pro-inflammatory markers (Braitch et al., 2009), and potential modulation by the ADNP derived drug candidate davunetide (NAP).

While VIP may present a dual function in the regulation of multiple sclerosis (Abad et al., 2010; Loh et al., 2011), it should be borne in mind that ADNP regulation, while in part associated with VIP and PACAP, is regulated by other control mechanisms. While VIP knockout exhibited a subtle phenotype (e.g., Abad et al., 2010; Loh et al., 2011) and VIP over expression, eventually led to down-regulation of activity with learning deficits (Gozes et al., 1993), ADNP knockout is lethal at embryonic time of brain formation, implicating ADNP as crucial for brain formation (Pinhasov et al., 2003; Mandel et al., 2007). Interestingly, VIP is suggested as a growth factor to the developing embryo (Gressens et al., 1993, 1994). Furthermore, partial ADNP deficiency leads to cognitive and social dysfunction (Vulih-Shultzman et al., 2007), similar to partial deficiency in VIP functions (Glowa et al., 1992; Gozes et al., 1993; Hill et al., 2007). This deficiency can be also associated with deficiency at the cellular level, with ADNP knockdown (or partial deficiency) associated with blocked neurite outgrowth (Mandel et al., 2008) and reduced glial neurotrophic milieu (Pascual and Guerri, 2007; Vulih-Shultzman et al., 2007) and with VIP blockade resulting in neuronal damage (Hill et al., 1994) and inhibition of synaptogenesis (Blondel et al., 2000).

In the case of ADNP, davunetide, an eight amino acid peptide snippet of ADNP is in phase 2/3 clinical trials in a severe neurodegeneration, progressive supranuclear palsy (PSP) (Gold et al., 2012).

## Future perspective

While this review focused on VIP and ADNP, from my point of view, it should be taken into consideration that we have developed a family of peptide hybrids/fragments and lipophilic VIP analogs, agonists and antagonists (Gozes et al., 1995), with activities ranging from neurtrophism/neurodevelopment neuroprotection (Gozes et al., 1999) to stimulation of sexual/social function and cancer growth inhibition. The precise GPCR involvement in these analogues activities remains to be elucidated (Gourlet et al., 1998; Moody et al., 2002). Interestingly, ADNP expression was shown to be reduced as a consequence of ischemia in tissue culture, and this was inhibited by treatment with the neuroprotective lypophilic VIP analog Stearyl-Norleucine17 VIP(7–28) (SNV) (Sigalov et al., 2000), awaiting future development.
